# General design flow for waveguide Bragg gratings

**DOI:** 10.1515/nanoph-2024-0498

**Published:** 2025-01-28

**Authors:** Frank Brückerhoff-Plückelmann, Tim Buskasper, Julius Römer, Linus Krämer, Bilal Malik, Liam McRae, Linus Kürpick, Simon Palitza, Carsten Schuck, Wolfram Pernice

**Affiliations:** Center for NanoTechnology (CeNTech), Heisenbergstr. 11, 48149 Münster, Germany; Kirchoff-Institute for Physics, University of Heidelberg, Im Neuenheimer Feld 227, 69120 Heidelberg, Germany; Center for Soft Nanoscience (SoN), Busso-Peus-Str. 10, 48149 Münster, Germany

**Keywords:** waveguide Bragg gratings, integrated signal processing, photonic longpass filter

## Abstract

Bragg gratings are crucial components in passive photonic signal processing, with wide-ranging applications including biosensing, pulse compression, photonic computing, and addressing. However, the design of integrated waveguide Bragg gratings (WBGs) for arbitrary wavelengths presents significant challenges, especially when dealing with highly asymmetric layer stacks and large refractive index contrasts. Convenient approximations used for fiber Bragg gratings generally break down in these cases, resulting in nontrivial design challenges. In this work, we introduce a general simulation and design framework for WBGs, which combines coupled mode theory with three-dimensional finite-element method eigenfrequency computations. This approach allows for precise design and optimization of WBGs across a broad range of device layer stacks. The design flow is applicable to further layer stacks across nearly all wavelengths of interest, given that the coupling between the forward and backward propagating mode is dominant.

## 1 Introduction

Bragg gratings are extremely versatile passive photonic devices due to their tailorable spectral response. Since the spectral response depends on the optical properties of waveguide material, fiber Bragg gratings are investigated for sensing various parameters such as temperature, strain, or acceleration [1], [2] and also deployed in commercial applications including power plants [3]. Integrating the Bragg gratings on-chip and shrinking down the size of the waveguide cross section [4], [5] increases the sensitivity to the surrounding media due to the evanescent field of the guided modes, making the gratings well suited for chemical sensors [6], [7]. Besides sensing, Bragg gratings also offer unique possibilities for photonic pulse manipulation, optical data transmission, and photonic computing. Chirped gratings, i.e., Bragg gratings with a varying periodicity along the propagation direction, can restore pulse shapes that are distorted by group velocity dispersion in fibers and compress pulses [8], [9], [10]. Similarly, they provide possibilities for dispersion engineering of frequency combs [11], [12] and offer a means to improve photonic computing systems based on frequency time interleaving [13]. In addition, Bragg gratings can reflect predefined frequency bands and thus be implemented as optical filters and multiplexers. Especially intriguing is the design of ultra-high extinction ratio Bragg filters for pump light suppression for quantum applications [14], [15], [16]. Here, first designs achieve suppression by more than 60 dB while featuring an insertion loss below −1 dB [17], [18].

Coupled mode theory (CMT) combined with the layer peeling/layer adding method is a convenient toolkit to design arbitrary fiber Bragg gratings as the modulation strength of the grating is small, and only coupling between the forward and backward propagating mode needs to be considered [19]. However, convenient approximations, particularly the estimate of the coupling coefficients directly from the strength of the effective index modulation [20], are only partially applicable and fail entirely for use cases with high mode confinement and refractive index contrast, e.g., when observing zero photonic band gaps for silicon waveguide Bragg gratings [21]. To circumvent those limitations, the coupling coefficients can directly be extracted from measured or simulated reflection spectra at the expense of significant computational overhead [22].

Herein, we present a general design flow for waveguide Bragg gratings (WBGs) that is compatible with CMT for nonuniform grating design and deploys 3d finite element method simulations with periodic boundary conditions as a computationally cheap way to extract the coupling coefficients for the parametrized grating cells. We illustrate the flexibility of the design flow by fabricating WBGs in 330 nm silicon nitride at telecom wavelengths and in 100 nm tantalum pentoxide for visible light. Furthermore, we use the eigenfrequency analysis to investigate the coupling to radiative modes in order to implement a photonic longpass filter in the visible.

## 2 Simulation and design framework

**Figure 1:**
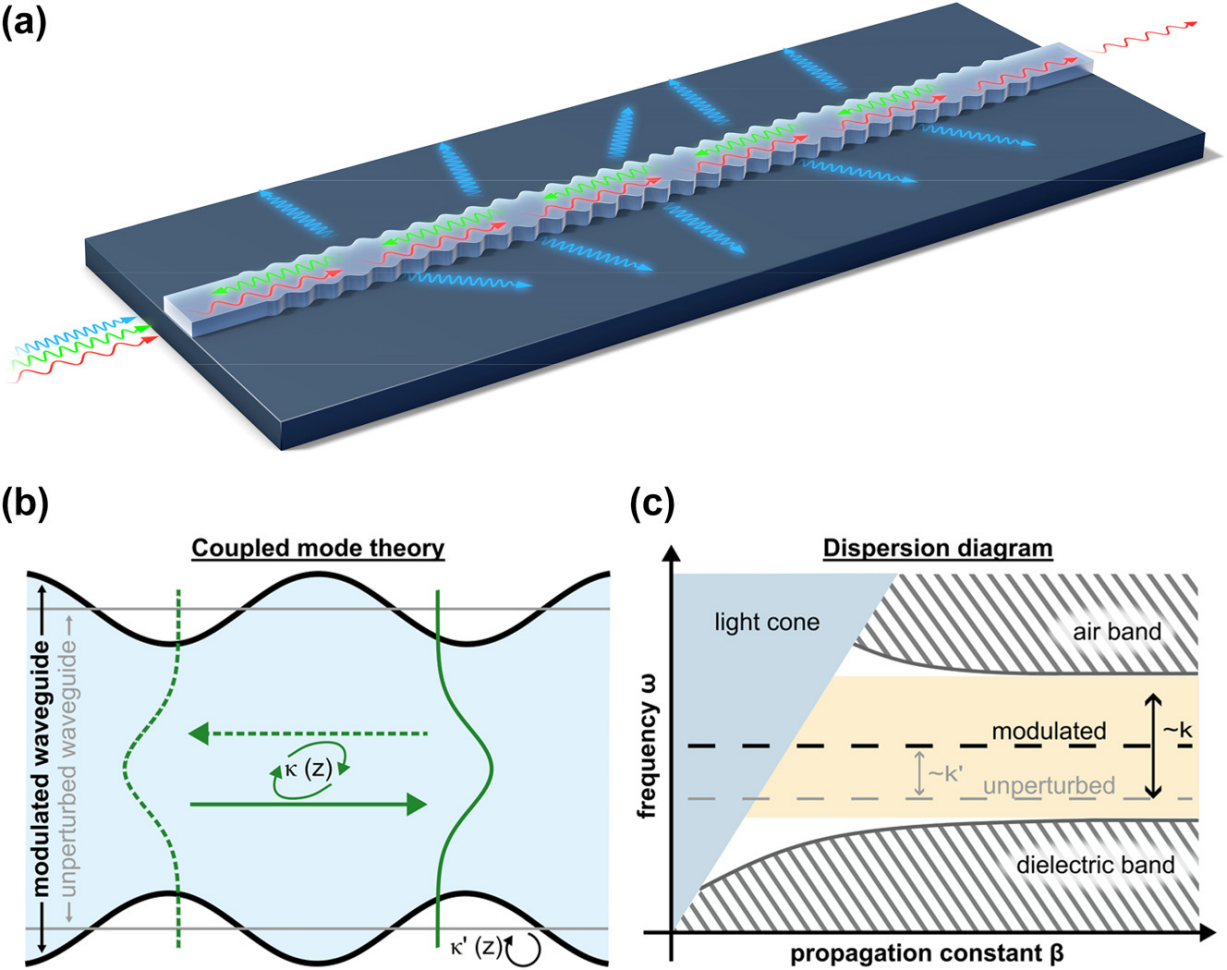
Waves in periodic structures. (a) The periodic modulation of the waveguide structure enables molding the flow of light. The spectral response depends on the frequency and effective index of the electromagnetic waves and can lead to scattering to the cladding or substrate, back reflection in the waveguide or unaffected transmission through the waveguide. (b) Coupled mode theory offers an effective way to simulate and design the propagation properties of a waveguide Bragg grating by expanding the modes of the structure in terms of eigenmodes of the unperturbed waveguide. It is often sufficient to only consider coupling between the forward and backward propagating fundamental mode. (c) The dispersion diagram for a uniform, infinite long periodic structure offer additional insight in the propagation properties. The modulation splits the fundamental mode into a dielectric band and air band, separated by a photonic band gap. Furthermore, the light line indicates the scattering into the substrate or cladding.

The response of an electromagnetic wave on a periodically distributed medium depends on the ratio between the effective wavelength along the direction of periodicity and the periodicity itself. As Figure 1a indicates, three qualitatively different response regions exist for the on-chip WBG. If the effective wavelength is approximately twice the periodicity, reflected waves constructively interfere, thus leading to an increase in reflected power [17]. If the effective wavelength becomes larger, the wave does not fully resolve the modulation anymore. Hence, the transmission is increased, enabling the design of subwavelength gratings [23]. In contrast, if the effective wavelength becomes smaller than the resonance wavelength, the wave starts to resolve the periodic modulation and induce scattering to the substrate and cladding, correspondingly the basis for grating couplers [24]. Therefore, the validity of simulation methods, and especially their underlying assumptions, strongly depends on the use case.

Here, we introduce a simulation and design framework for WBGs that combines coupled mode theory with an eigenfrequency analysis of parametrized grating cells. Coupled mode theory is a powerful method to describe the propagation within the modulated waveguide. Even though the eigenmodes of the unperturbed waveguide are not solutions of the Maxwell-equations for the perturbed structure, they form a complete basis together with the continuum states [25]. Thus, we can expand the wave in terms of the unperturbed eigenmodes and compute the coupling coefficients between each via overlap integrals. To decrease the computational costs, it is reasonable to only consider coupling between the forward propagating and backward propagation fundamental modes for weak perturbations and frequencies close to the photonic bandgap as shown in Figure 1b. One main advantage of CMT is that it is compatible with varying coupling coefficients along the propagation direction, thus enabling the simulation of nonuniform grating designs. On the other hand, we can exploit periodic boundary conditions to precisely compute the eigenfrequencies of a single grating cell and thus obtain the dispersion diagram for an infinitely long uniform WBG, as sketched in Figure 1c. The periodic perturbation of the waveguide induces a photonic bandgap between the fundamental dielectric and air band of guided modes. Within the bandgap, no propagating guided modes exist, i.e., the propagation vector would be imaginary.

We design the WBG by deploying CMT to model the response of nonuniform gratings but use the eigenfrequency analysis to compute the coupling coefficients for the individual grating cells. The mapping between the coupling coefficients and the eigenfrequency analysis is done as follows: For a uniform grating and only considering coupling between the forward and backward propagating fundamental mode with amplitudes a(z) and b(z), respectively, we can write the CMT equations as [19]:

(1) $$\frac{d}{dz}\left(\begin{array}{c} a\left(z\right)\\ b\left(z\right) \end{array}\right)=-i\left(\begin{array}{cc} \Delta \beta & \kappa \\ -\kappa & -\Delta \beta \end{array}\right)\cdot \left(\begin{array}{c} a\left(z\right)\\ b\left(z\right) \end{array}\right)$$

Here, the time dependency is neglected. The parameter Δ*β* = *β*_*u*_ + *κ*′ + *π*/Λ is the phase detuning factor, *β*_*u*_ is the propagation constant of the unperturbed waveguide, *κ*′ the self-coupling coefficient, *κ* is the coupling coefficient given by the grating strength, and Λ the periodicity of the grating. For the forward propagating mode, we find with the boundary condition $a\left(0\right)=1,b\left(L\right)=0$ , where L is the length of the grating:

(2) $$a\left(z\right)={\text{e}}^{\sigma z},\;\sigma =\sqrt{\kappa^{2}-\Delta \beta^{2}}$$

Thus, the wave exponentially decays along the grating direction within an area around the center wavelength:

(3) $$\lambda_{\text{Bragg}}=\frac{2\pi n_{\mathrm{eff}}\Lambda }{\pi -\kappa^{\prime }\Lambda },\;BW=\frac{\lambda_{\text{Bragg}}^{2}\kappa }{\pi n_{g}}$$

Here, *n*_*g*_ is the group index of the unperturbed waveguide’s ground mode at the center wavelength of the grating *λ*_Bragg_. As we can extract the exact position $\left(\lambda_{\text{Bragg}}\right)$ and the width $\left(BW\right)$ from the photonic bandgap, we can compute it via the eigenmode analysis. Note that the term *n*_eff_Λ, *n*_eff_ is the effective refractive index of the considered fundamental mode, also directly follows from the eigenmode analysis for the case of zero amplitude modulation.

**Figure 2:**
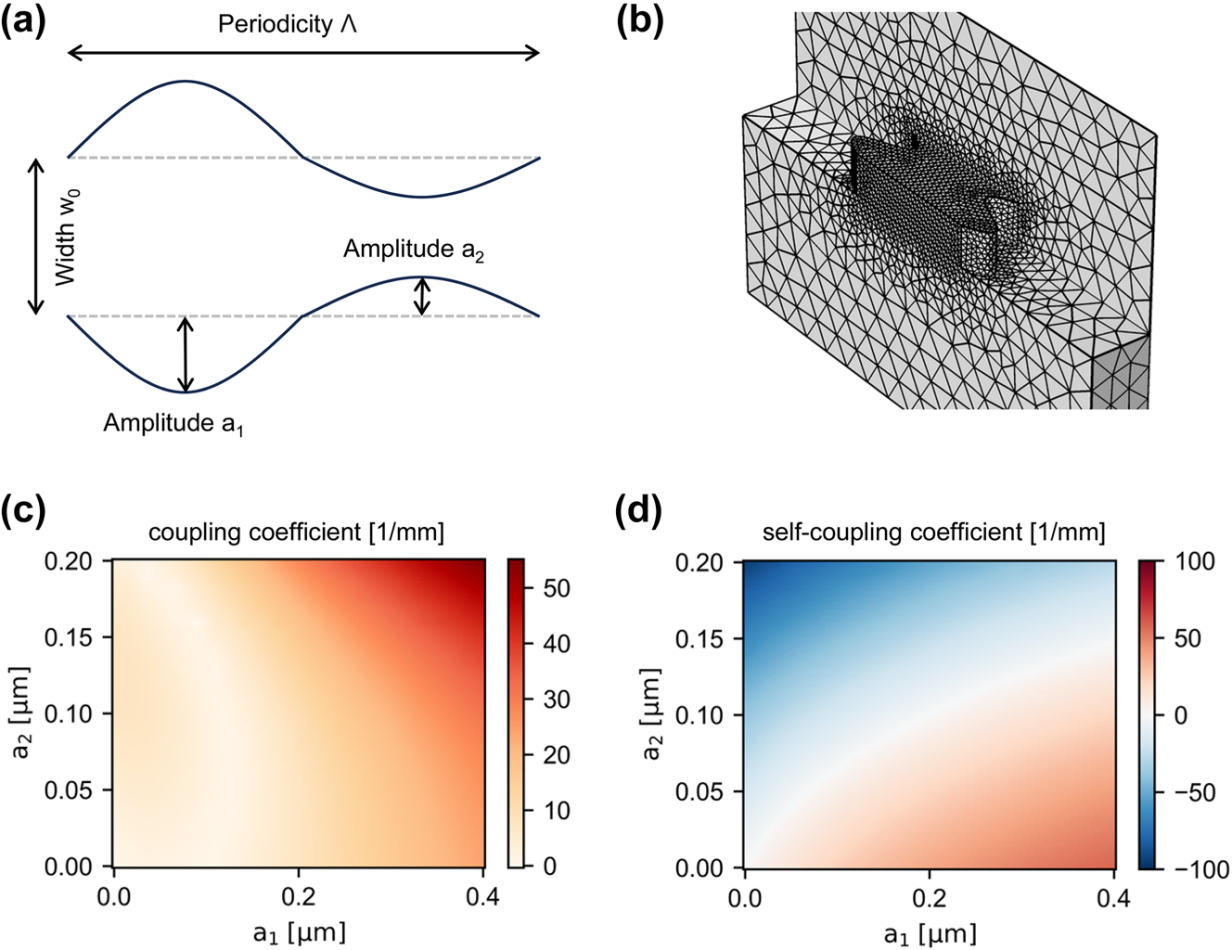
Simulation and design framework. (a) We design the grating cell as a sine modulation of the waveguide width, where the modulation from outside to inside and vice versa are tuned independently via the amplitudes a_1_ and a_2_. The waveguide width is set to the default width of the photonic circuit, and the periodicity determines the general spectral response. (b) For a given periodicity and width, we compute the eigenfrequency of the grating cell for various values of a_1_ and a_2_ at the edge of the Brillouin zone *β* = *π*/Λ. As the simulation domain only contains a single grating cell, complete finite element method simulations with a fine mesh can be performed in reasonable time. (c, d) Based on the FEM simulations, we can compute the width and position of the bandgap and hence the coupling coefficient for the coupled mode theory approximation.

Figure 2 shows the workflow for WBG design for arbitrary material stacks and wavelength, here, representatively for 330 nm thick silicon nitride waveguides on silicon dioxide without cladding. First, we divide the overall grating into individual grating cells and design each of them according to Figure 2a. The shape of a waveguide with width w_0_ is modulated by two half sine-curves with amplitudes a_1_ and a_2_. The length of the grating cell is given by the periodicity Λ. To extract the coupling coefficients, we only need to compute the eigenfrequencies of the grating at the edge of the Brillouin zone, i.e., the width and position of the photonic bandgap. Thus, we can conveniently use periodic boundary conditions and set the wavevector component along the propagation direction to Λ/*π*. Due to the small scale of the simulation, we can perform 3d finite element method (FEM) simulations with fine mesh settings as shown in Figure 2b. In this way, we directly include fabrication imperfections such as shrinkage of the mask or potential sidewall angles. We perform the eigenfrequency analysis with Comsol using periodic boundary conditions for the front and end facet of the grating cell and scattering boundary conditions for the four side walls. To obtain optimal results with the periodic boundary conditions, it is crucial that not only the geometry but also the mesh is identical at the front and end facet. Thus, we create a 2d mesh for the front, copy the exact mesh to the end facet, and use Comsols automatic 3d meshing for the rest of the structure. The maximal mesh size is 20 nm within the waveguide material and 100 nm for the SiO_2_ and air.

Figure 2c shows the coupling coefficient extracted from the simulation for grating cells with a periodicity of 500 nm, an unperturbed designed width of 1.2 μm, and resist shrinkage to 98 % of the designed width. One directly observes that the properties of a Bragg grating in the highly asymmetric, large refractive index material stack with large refractive index contrast do not follow the general approximations applicable in fiber optics or weakly guided systems. For instance, the coupling coefficient is not proportional to the (effective) refractive index modulation [20] and does not strictly increase with increasing modulation strength. In contrast, we obtain a zero photonic bandgap for a set of modulation amplitudes corresponding to the white line in Figure 2c, which agrees with observations for silicon WBGs [21]. Furthermore, the self-coupling coefficients also show a nontrivial dependency on the amplitudes of the modulation. Therefore, we use CMT in the approximation of single-mode coupling to obtain the profile of coupling coefficients and self-coupling coefficients while we apply the 3d FEM eigenfrequency analysis to map the coupling coefficients to physical design parameters.

## 3 Telecom notch filter

**Figure 3:**
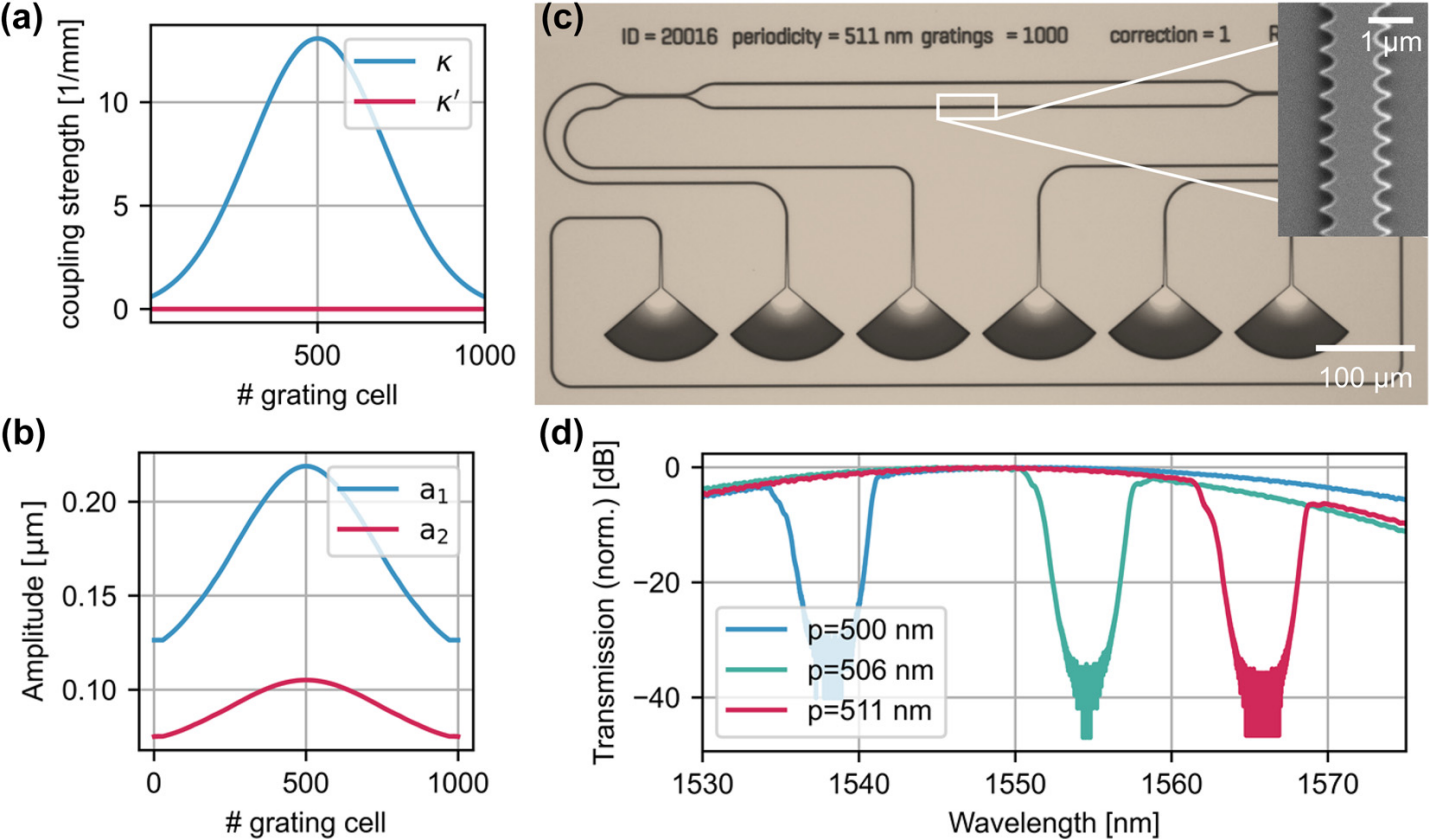
Apodized waveguide Bragg gratings. (a) In order to fabricate a 5 nm notch filter with suppressed sidelobes, we apodize the coupling coefficient with a Gaussian window. The self-coupling coefficient needs to remain constant, in this case zero, to avoid asymmetric reflection spectra. (b) We use the FEM simulations to map the coupling coefficients to the physical design parameters of the grating cell. (c) We fabricate air-clad waveguides and filters on the 330 nm silicon platform. We place two identical WBGs in a Mach–Zehnder Interferometer-like structure to separate the reflected light from the input light. (d) We measure the transmission spectrum for three different gratings with a periodicity of 500 nm, 506 nm, and 511 nm, respectively. We can use the same FEM simulation, performed for a periodicity of 500 nm, for all designs as the coupling coefficients are invariant for small variations of the periodicity.

We fabricate WBG based on the previous simulations, deploying ARN resist and a Raith EBPG5150 for mask generation and reactive ion etching with CHF3 plasma generated in an Oxford PlasmaPro80 to transfer the pattern to the silicon nitride thin-film. Due to resist shrinkage, the fabricated waveguide width is about 2 % smaller than the designed one, which is considered in the simulations. We target a grating bandwidth of 5 nm and suppress grating sidelobes by apodizing the coupling coefficient with a Gaussian window, as shown in Figure 3a. In contrast, the self-coupling remains constant over the whole grating strength to avoid asymmetric reflection spectra [20]. The amplitudes a_1_ and a_2_ are simultaneously optimized to match the desired (self-) coupling coefficients, leading to the physical design parameters shown in Figure 3b. Note that due to the zero photonic bandgap, the ratio between the maximal and minimal amplitude is much smaller than the ratio between the maximal and minimal coupling coefficient.

Figure 3c shows a microscope image of the fabricated device. Two identical WBGs are placed in a Mach–Zehnder Interferometer (MZI)-like structure to separate the back reflected light from the input light [17], [26]. We fabricate three different gratings with identical amplitudes a_1_ and a_2_ but different periodicities, namely 500 nm, as in the simulation, 506 nm and 511 nm. We fabricate the 500 nm device in a different fabrication run to gauge the robustness of the design flow. We characterize the gratings by measuring the wavelength-dependent transmission using a tunable Santec TSL-770 laser and two Newport Model 2011 photodetectors. Figure 3d shows the transmission spectra normalized to the maximum transmission. The center wavelength increases from 1,538.2 to 1,565.6 nm with the periodicity of the grating and is robust with respect to the (in-house) fabrication process. Independent from the periodicity, the sidelobes of the notch filters are suppressed, and the extinction ratio is around 30 dB, highlighting the robustness of the design flow. The extinction ratio is limited by back reflections, from the fiber array facet and grating couplers, and phase errors within the gratings arising from both fabrication errors and potential design errors due to the mismatch between the shrinkage model and the physical shrinkage. By cascading decoupled gratings, the extinction ratio can be increased [17].

## 4 Visible longpass filter

Operating the Bragg grating close to its photonic bandgap mainly induces coupling to a discrete set of guided modes as for example shown in Figure 3d. As phase-matching is required for constructive interference, this process highly depends on the wavelength and especially the modes’ effective index. In contrast, we construct a longpass filter by choosing design parameters such that undesired frequencies lie within the light cone. Therefore, they scatter to the continuous set of unbound substrate and cladding modes, enabling broadband, mode-insensitive filtering. As an example, we set the cutoff wavelength of the TE 0 like modes to 690 nm, since a typical wavelength for the off-resonantly excitation of solid-state emitters is 633 nm (HeNe Laser) and use the 100 nm tantalum pentoxide (TaO) on-insulator platform. TaO is a promising platform for integrated quantum photonics since it is transparent over a broad spectral range [27], has low propagation loss [28], exhibits low autofluorescence [29], facilitating single-photon emitter integration [30], [31], and has a large third-order optical nonlinearity [32].

**Figure 4:**
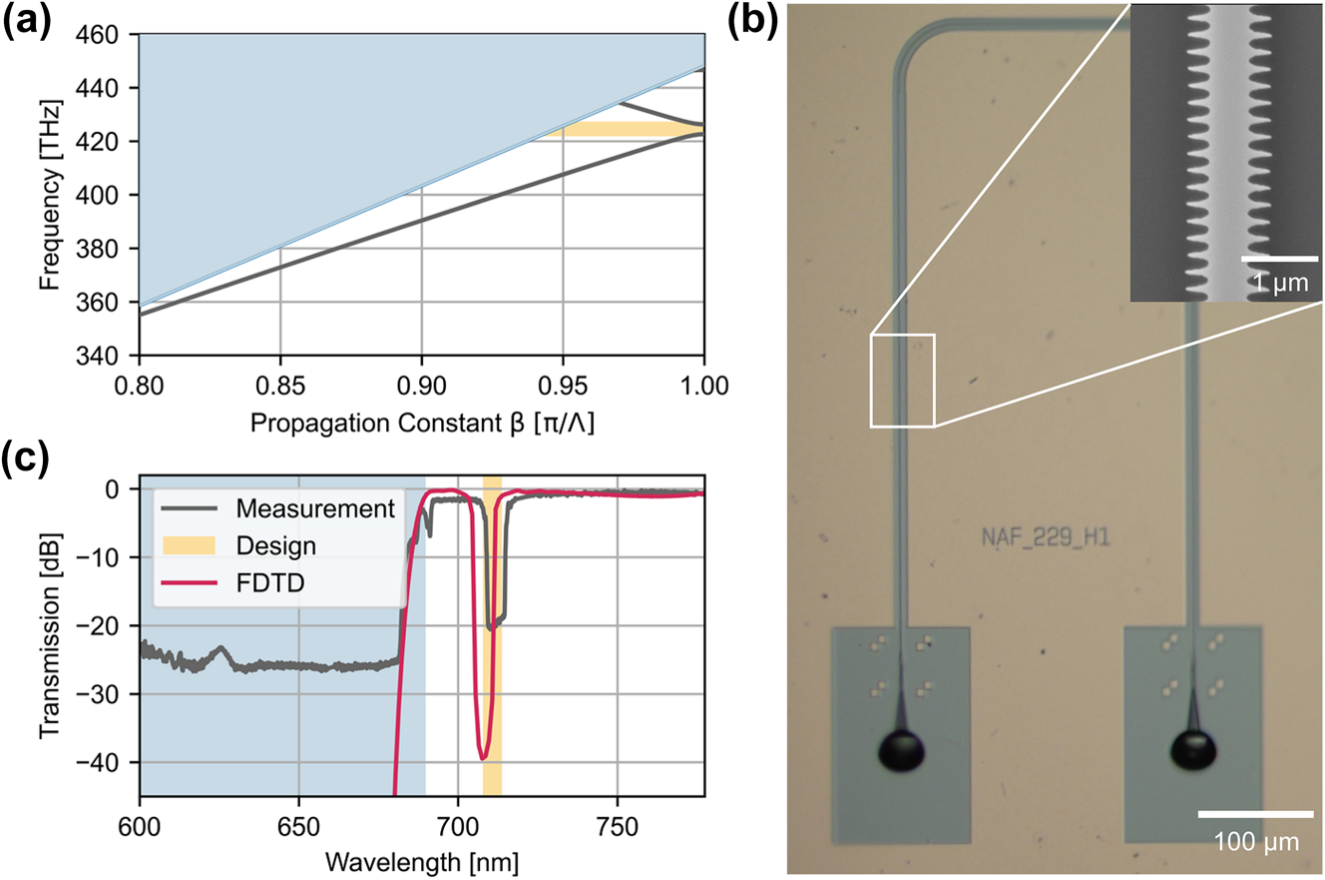
Broadband visible filter. (a) The dispersion diagram shows the dielectric and air band of the fundamental mode separated by the band gap (yellow-shaded region), the dielectric band of the first-order mode and the light cone (blue-shaded region). The periodic modulation of the waveguide structure leads to significant coupling to substrate modes if no guided mode exists for the given frequency. (b) We fabricate a Bragg grating with 2000 elements and a periodicity of 229 nm on the 100 nm TaO platform that implements a longpass filter. The SEM image shows a single grating cell of the structure. We achieve broadband coupling to the photonic circuit by deploying 3d-printed total internal reflection couplers. (c) We measure the transmission spectrum of the low-cut filter normalized to the back loop reference transmission. For small wavelengths, the coupling to unbound modes decreases the transmission by about −25 dB. The loss within the dielectric band is as low as −0.4 ± 0.1 dB.

We deploy the simulation framework explained in Figure 2 to design a unit cell with a periodicity of 229 nm and a waveguide width of 700 nm. We set the modulation strength to a_1_ = 290 nm and a_2_ = 81 nm to obtain zero self-coupling and a bandgap of 6 nm. Figure 4a shows the simulated dispersion diagram for the grating cell. There are guided modes in the TE0 like dielectric and the air band separated by the photonic bandgap (yellow-shaded region) and guided modes in the TE1 like dielectric band very close to the light cone. Apart from guided modes, there is a continuum of unbound modes in the light cone (blue-shaded region). By exploiting that there is no guided TE0 like modes above 434.6 THz (690 nm), we implement a photonic longpass filter. Qualitatively, this regime is the opposite of the subwavelength regime. The wavelength of the electromagnetic wave becomes so small that it starts to resolve the modulation of the waveguide width and, hence, is substantially affected by scattering.

We fabricate a 2000 element uniform grating on TaO by using a Raith EBPG5150 in combination with the positive resist AR-P 6200.04 to pattern the mask and deploy reactive ion etching in a CHF3/Ar/CF4 plasma generated by an Oxford PlasmaPro 80 to transfer the mask to the chip. Figure 4b shows an optical microscope image of the fabricated device and an SEM image of the grating itself. 3d-printed polymer couplers based on total internal reflection enable broadband coupling to the photonic circuit [33]. We measure the transmission spectrum with a Zolix spectrograph with an Andor Newton camera and NKT Photonics SuperK COMPACT white light source and normalize the counts to a 310 μm long reference back loop. We compare the measured spectrum with the designed features obtained from 3d FEM simulation and the result of the commercially available Lumerical 3d FDTD solver. We deploy perfectly matched layer boundary conditions in the FDTD simulation to mitigate any nonphysical behavior of the boundaries due to light scattering and perform the simulation for the full grating design instead of exploiting periodic boundary conditions. The designed features of the low-cut filter are evident in Figure 4c. First, there is a drop in transmission at 692 nm, as there is no guided TE0-like mode anymore in the structure. Next, the transmission decreases to the noise floor of around −25 dB for wavelengths below 681.5 nm (blue-shaded region). The noise floor of −25 dB is likely due to coupling from substrate modes back to the unperturbed waveguide behind the grating. Due to the grating itself, we also see the photonic bandgap (yellow-shaded region) in the transmission spectrum. As the light is reflected back in the waveguide and not scattered out, the filtering is significantly more phase-sensitive, thus limiting the extinction ratio by phase errors in the fabricated structure. The loss of the longpass filter is as small as −0.4 ± 0.1 dB in the dielectric band.

## 5 Discussion

The presented design flow provides a robust framework for the fabrication of Bragg gratings for arbitrary wavelengths as well as highly asymmetric layer stacks and large refractive index contrasts. Due to the link between coupled mode theory and eigenfrequency analysis, we can determine coupling coefficients in a computationally cheap way and furthermore avoid regions of abnormal behavior, e.g., zero photonic bandgap phenomena. We demonstrated its application on nonuniform apodized gratings and showed the periodicity independence of the filter characteristics in a range of 30 nm around 1,550 nm. Thus, designing chirped gratings with varying periodicity offers an intriguing possibility for pulse shape manipulation and photonic computing without requiring additional eigenfrequency computations. Going one step further, it would be intriguing to obtain coupling coefficients for arbitrary filter responses using the Layer Adding/Layer Peeling algorithm in a first step [26], [34], [35], followed by creating a suitable physical grating on an arbitrary platform by employing the provided design flow.

The ability to include fabrication imperfections such as shrinkage in a computational cheap way is powerful, especially for designing multichannel wavelength multiplexers as in our previous work for quantum key distribution [36] and photonic neuromorphic computing [17]. In general, uniform gratings are more robust to (2d systematic) fabrication errors as all grating cells are impacted identically. Thus, there is a constant offset in the resonance wavelength, which will be small in comparison to the impact of vertical tolerances like variations in the waver thickness in most cases. The offset can be easily compensated for by changing the periodicity without requiring rerunning the FEM simulation. Also, the width of the bandgap might be impacted. However, for a bandwidth on a nm scale, a significant error would require a mismatch between designed and simulated amplitudes of several 10 nm as shown in Figure 2c. For apodized gratings, each grating cell has different amplitudes and thus has a different mismatch between designed and fabricated coupling coefficients. If the gradients of the coupling coefficients with respect to the amplitudes are similar for all gratings cells, i.e., especially avoiding the zero photonic bandgap region, the impact on the shape of the transmission spectrum is likely negligible. Otherwise, the parameterization offers a direct way for on-chip parameter sweeps by applying different transforms to the coupling coefficients during the GDS design.

Furthermore, we investigated a grating designed for coupling to radiative modes for high frequencies while providing low insertion loss for lower frequencies in the dielectric band at visible wavelengths. To further increase the extinction ratio, coupling from substrate modes back to the waveguides must be avoided. As the light is scattered out of the waveguide for high frequencies and is not back-reflected, one might adopt the scheme for cascading the WBGs presented in [18]. Here, several short filters connected via waveguide bends are deployed instead of one longer one. These bends reduce the impact of substrate modes’ scattering and consequently enhance the extinction ratio. In addition, absorptive materials can be placed in the vicinity of the waveguide to suppress substrate modes.

Broadband visible filters might be especially intriguing in combination with solid-state single-photon emitters as they can be off-resonantly excited by a laser, emitting photons during deexcitation. However, subsequently, the laser light and the emerging autofluorescence of the material, which covers a large spectral range, need to be filtered out without interfering with the light emitted from the single-photon emitter. The presented low-loss integrated longpass filter is an alternative to off-chip filters that utilize different dielectric-coated spectral filters and pave the way toward fully integrated quantum systems [14], [15], [16].
